# Evaluation of a trauma team activation protocol revision: a prospective cohort study

**DOI:** 10.1186/s13049-016-0295-3

**Published:** 2016-08-25

**Authors:** Trond Dehli, Svein Arne Monsen, Knut Fredriksen, Kristian Bartnes

**Affiliations:** 1 Department of Gastrointestinal Surgery, University Hospital North Norway (UNN), 9038 Tromsø, Norway; 2 Department of Clinical Medicine, UiT- The Arctic University of Norway, 9037 Tromsø, Norway; 3 Department of Anesthesiology, Helgeland Hospital, 8801 Sandnessjøen, Norway; 4 Division of Emergency Medical Services, UNN, 9038 Tromsø, Norway; 5 Department of Cardiothoracic and Vascular Surgery, UNN, 9038 Tromsø, Norway

**Keywords:** Trauma, Triage, Patient transfer, Emergency treatment

## Abstract

**Background:**

Correct triage based on prehospital information contributes to a better outcome for potentially seriously injured patients. In 2011 we changed the trauma team activation (TTA) criteria in our center in order to improve the high over- and undertriage properties of the protocol. Five criteria that were unable to predict severe injury were removed. In the present study, we evaluated the protocol revision by comparing over- and undertriage in the former and present set of criteria.

**Methods:**

All severely injured patients (Injury Severity Score (ISS) > 15) and all patients admitted with TTA in the period of 01.01.2013 – 31.12.2014 were included in the study. We defined overtriage as the fraction of patients with TTA when ISS ≤15 and undertriage as the fraction of patients without TTA when ISS > 15. We also evaluated triage with the occurrence of emergency procedures immediately after admission.

**Results:**

324 patients were included, 164 patients had ISS>15, 287 were admitted with TTA. Over- and undertriage were 74 % and 28 % respectively. When we used emergency procedure as reference, the figures were 83 % and 15 % respectively. Undertriaged patients had significantly more neurosurgical injuries and were significantly more often transferred from an acute care hospital.

**Discussion:**

Over- and undertriage are almost the same as before the criteria were revised, and higher thanrecommended levels.

**Conclusions:**

Revision of the TTA criteria has not improved triage, and further measures are necessary to achieveacceptable levels.

## Background

Multidisciplinary trauma teams are important in trauma care by allowing timely diagnosis and treatment of unstable trauma patients [1]. Criteria-based trauma team activation (TTA) protocols are widely implemented throughout Scandinavia [2]. TTA criteria vary among trauma centers, but they usually resemble the recommendations from the American College of Surgery – Committee on Trauma (ACS-COT) [3–7]. The criteria comprise parameters of physiologic compromise, anatomic injury, and mechanism of injury (MOI), and TTA is recommended when prehospital information indicates that at least one criterion is fulfilled.

A substantial overtriage (TTA despite only minor or moderate injury) is common and may reach 70 % in some centers, mostly reflecting a low specificity of the MOI criteria [5–7]. Overtriage is mainly a resource problem, as it diverts personnel from other activities in the hospital. However, undertriage (admission of severely injured without TTA) may delay timely intervention and even increase mortality [8]. Some overtriage seems to be necessary to avoid unacceptable undertriage, and the ACS-COT suggests that 50 % may be acceptable [3]. However, no scientific evidence supports this figure, and it may depend on factors related to the individual facility and trauma system. Injury patterns vary considerably between regions, and the predictive properties of triage criteria depend on the incidence of severe injuries. In Scandinavia penetrating injuries are rare [9, 10], and the frequency of severe polytrauma admissions is low, except in some large cities [11].

In order to optimize patient outcomes and resource utilization we evaluated the TTA protocol of the University Hospital of North Norway Tromsø (UNN) in 2011 using data from the period 2005–6 [12]. Five criteria were removed from the TTA-protocol. In the present study we evaluate the impact of the TTA protocol revision on protocol performance of under- and overtriage.

## Methods

### Design

The study is a prospective cohort study based on the trauma registry at UNN.

### Study setting and local trauma registry

UNN has a primary catchment population of 80.000, and is the regional trauma center (level I-II according to ACS-COT) for 480.000 people. The mainland covers an area of 107 000 km^2^, and is the same length as the British Isles from south to north, with 9 acute care hospitals. The closest acute care hospital is 250 km from the trauma center by road; therefore all patient transfers between hospitals are done with air ambulance. According to the regional health trust’s destination and transfer protocol, patients with injuries that exceed the resources of the acute care hospitals will be transferred to the UNN either directly from the scene or from the local hospital after initial stabilization. The emergency medical communication center (EMCC) at UNN mobilizes the trauma team before patient arrival based on defined TTA criteria (Table 1). Trauma patient transferred to the UNN are admitted with TTA if there was TTA at the local hospital and if they arrive within 24 h after the injury.

**Table 1:** An analysis of individual criteria applied for trauma team activation

Criteria category	Criterion	Criterio n(/reason) applied to the patient (no. of patients)	Criterion applied to a severely injured patient (ISS > 15^a^), (no. of patients)	Criterion applied to a patient receiving an emergency procedure (no. of patients)
Vital parameters	1. Airway obstruction, stridor	4	2	3
	2. Tachypnoe (adults, respiratory rate > 30)	14	10	3
	3. Heart rate > 130 (adults)	3	0	2
	4. Systolic BP <90 mmHg	9	5	4
	5. Lowered level of consciousness (GCS^b^ <13)	87	33	28
Extent of injuries	6. Flail chest	2	1	0
	7. Unstable fracture of the pelvis. Fracture in two or more long bones	5	2	0
	8. Traumatic amputation or crush injury above wrist/ankle	1	0	1
	9. Injury in two or more body regions (head/neck/chest/abdomen/pelvis/femur/back)	61	9	8
	10. Paralysis	10	8	1
	11. Penetrating injury of the head/neck/chest/abdomen/pelvis/groin/back	5	0	3
	12. 2. or 3. degree burn injury > 15 % body surface (children > 10 %)	5	2	3
	13. Burn injury with inhalation injury	5	2	2
	14. Hypothermia (core temperature <32 °C)	11	3	2
Mechanism of injury	15. Ejected from vehicle	6	4	0
	16. Co-passenger dead	5	2	0
	17. Trapped in wreck	9	3	1
	18. Pedestrian or cyclist hit by motor vehicle	15	2	2
	19. Fall from >5 m	20	10	3
	20. Avalanche accident	1	0	0
Unknown criteria	ETA^c^ < 15 min	8	0	0
	Trauma team leader requested TTA^d^	5	0	0
	Anesthesiologist in ambulance helicopter requested TTA^d^	6	0	0
	Unknown/undocumented reason for TTA^d^	20	0	0

The table shows the number of times an individual criteria is applied for trauma team activation based on prehospital information in potentially severely injured patients primarily admitted at the University Hospital of North Norway Tromsø during 2013–14, *n* = 223. Transferred patients are not included in this analysis. The two last columns shows the number of times the individual criterion correctly activated the trauma team assessed with ISS and the appearance of an emergency procedure. More than one criterion can be applied to one patient

Emergency surgical procedure include endotracheal intubation, damage control thoracotomy, damage control laparotomy, extraperitoneal packing in the pelvis, revascularization of an extremity, intervention radiology, craniotomy, insertion of intracranial pressure bolt, chest tube insertion, external fracture stabilization or other emergency procedures aiming at stabilizing airway, respiration or circulation

ISS^a^: Injury Severity Score, ISS > 15: Severely injured patient, GCS^b^: Glasgow Coma Score, ETA^c^: estimated time of arrival, TTA^d^: trauma team activation

The trauma registry at UNN includes patients admitted with TTA, patients with penetrating injury to the head/neck/torso/extremities proximal for elbow or knee, all patients with New Injury Severity Score (NISS) >12 [13], and all patients with a head injury with abbreviated injury score (AIS) ≥3 [14]. The registry excludes patients with chronic subdural hematoma, with injuries from drowning, inhalation and strangulation, and those admitted for rehabilitation after trauma. Patients that did not have TTA but fulfilled the inclusion criteria were identified from the hospital admission charts. Trained and authorized registrars scored the AIS and calculated the ISS [14, 15].

### The revision of the UNN trauma team activation protocol

The former TTA protocol of the UNN was evaluated in 2011 based on data from the period 2005–6 [12]. The inclusion criteria were the same as in the present study. When we analyzed the data with an injury severity score (ISS) > 15 as indication of severe trauma [14, 15], the overtriage was as high as 71, and the undertriage was 32 %. With the occurrence of an emergency procedure as standard of reference, the overtriage was 71 and the undertriage 21 %. Three criteria in the mechanism of injury-category (“Motorcycle accident”, “Considerable deformation of vehicle compartment” and “Traffic accident with speed >60 km/h”) conferred a substantial overtriage and were removed from the TTA protocol. Another criterion was removed because it had not been used (“Convulsions”), and the criterion “Dilated or not responding pupils” was deemed unnecessary because the same patients were identified by “Lowered level of consciousness (GCS <13)”, both in order to simplify the criteria. The revised TTA criteria are listed in Table 1. The protocol also allows TTA if time to arrival of the injured patient <15 min and prehospital information on fulfillment of criteria is missing (e.g. immediate transport to the hospital is prioritized over examination). The 2011 study also showed that undertriaged patients were mainly patients who were transferred from local hospitals or patients admitted directly to the neurosurgical department. Based on the results from this study, the UNN also attempted to increase awareness of the inter-hospital transfer protocol among employees in the EMCC, in the neurosurgical department, and among trauma team leaders.

### Inclusion of patients

In the present study, we included all patients admitted to the UNN with TTA and all patients with an ISS > 15 (with or without TTA) during the period 1.1.2013 – 31.12.2014. Patients transferred >24 h after injury were excluded.

### Data collection

Most data could be collected from the trauma registry, but TTA criteria were recorded from the EMCC record AMIS (acute medical communication system, Nirvaco, Norway) and supplementary information was collected from the patient records of the UNN. Data from other hospitals were recorded from transfer documents.

### Triage

Triage was considered correct when the trauma team was mobilized for primary admittance of injured patients with ISS > 15, or an injured patient transferred to our trauma center < 24 h after injury and admitted with TTA in the local hospital. Overtriage was calculated as the proportion of TTA for patients with minor or moderate injuries (ISS ≤ 15). Undertriage was calculated as the proportion of severely injured patients (ISS > 15) admitted without TTA. The ability of an individual TTA criterion to predict appropriate TTA, was given as the number of patients that fulfilled the specific criterion that also had an ISS > 15. The same triage- and criterion analyzes are done with the occurrence of emergency procedures as standard of reference.

### Parameters

Baseline characteristics are listed in Table 2. The primary endpoint is TTA or no TTA for severely injured patients (ISS > 15), and the corresponding over- and undertriage.

**Table 2:** Main characteristics of patients received by a trauma team or having ISS^a^ > 15, admitted at the University Hospital of North Norway Tromsø in 2013–2014, *n* = 324

Male patients (proportion)	226 (69.8 %)
Mean age, years (range)	41 (0–101)
Median ISS* (interquartile range)	10 (2, 20)
Proportion with ISS > 15** (percentage of total)	131 (40.4 %)
Predominant mechanism of injury (proportion)	
Penetrating	3.4 %
Blunt	96.6 %
Mean length of stay (days)	6.7
Mean length of stay in intensive care unit (days)	2.0
Interhospital transfer, patients (n (proportion))	74 (22.8 %)
30 day mortality (n (proportion))	18 (5.6 %)
30 day mortality, patients with ISS > 15 (n (proportion))	17 (14.9 %)

ISS^a^: Injury Severity Score, ISS > 15: seriously injured patient

* Injury Severity Score

**Severely injured patient

Secondary outcome parameters include triage calculated with the occurrence of an emergency procedure as standard of reference, and the type and frequency of emergency procedures. Emergency procedures are listed in Table 3.

**Table 3:** Emergency procedures for a total of 324 trauma patients admitted with activation of the trauma team or ISS > 15* at the University Hospital of North Norway Tromsø during 2013–2014. One patient can receive more than one procedure, both in the local hospital and in the trauma center

Emergency procedure	Number of patients receiving a procedure among all patients (percentage of patients), *n* = 324	Number of patients receiving a procedure at the local hospital before transfer, *n* = 74	Number of undertriaged patients (no TTA** and ISS > 15) receiving a procedure, *n* = 37
Endotracheal intubation	57 (17 %)	27	-
Damage control thoracotomy	2 (0.6 %)	-	-
Damage control laparotomy	12 (3.7 %)	4	-
Extraperitoneal pelvic packing	1 (0.3 %)	-	-
Revascularization of an extremity	-	-	-
Intervention radiology	8 (2.5 %)	-	2
Craniotomy	26 (8.0 %)	-	8
insertion of intracranial pressure bolt	19 (5.9 %)	-	4
chest tube insertion	26 (8.0 %)	13	2
external fracture stabilization	7 (2.2 %)	2	2
Other procedures to stabilize airways, respiration or circulation	11 (3.4 %)	-	-
Sum of all patients with an emergency procedures	92	46	14

*ISS: Injury Severity Score, ISS > 15: seriously injured patient, **TTA: trauma team activation

### Statistical methods

Results are given as sum, percentage, mean and median with interquartile (IQR) range. Individual criteria which are seldom used (<5 of patients) and/or with a low positive predictive value (<10 %) will be considered omitted. Comparison of triage results with the 2011 study is done with Pearson correlation-test although data from the former study is not shown [12]. Characteristics of undertriaged patients are tested with Pearsson chi-square test. Significance is assumed for *p* < 0.05.

### Ethics and publication

The study was approved by the hospital’s data protection officer (case number 2013/501). Approval from the Regional Medical Research Ethical Committee was not necessary as the study was assessed as quality control and not medical research by the Ethical Committee itself (case number 2012/1912/REK Nord).

## Results

A total of 324 patients were included. Baseline characteristics are presented in Table 2. There were 131 patients with ISS > 15 and 94 of these (72 %) were admitted with TTA. Results for triage are presented in Tables 4 and 5. There were no significant changes in over- and undertriage of the TTA protocol following the revision after the 2011 study.

**Table 4:** Performance of the trauma team activation protocol during 2013–2014 at the University Hospital of North Norway Tromsø assessed with Injury Severity Score (ISS)

	Number of patients	Number of patients with ISS > 15	Correct triage (TTA^a^ and ISS >15)	Undertriage (no TTA^a^ and ISS > 15)	Overtriage (TTA^a^ and ISS < 15)
Primarily admitted patients	250	81 (32 %)	72 % (58/81)	28 % (23/81)	74 % (169/227)
Transferred patients	74	50 (68 %)	72 % (36/50)	28 % (14/50)	40 % (24/60)

TTA^a^: trauma team activation, ISS > 15: severely injured patient

**Table 5:** Performance of the trauma team activation protocol during 2013–2014 at the University Hospital of North Norway Tromsø assessed with the occurrence of emergency procedure

	Number of patients	Number of patients with emergency procedure	Correct triage (TTA^a^ and emergency procedure)	Undertriage (no TTA^a^ and emergency procedure)	Overtriage (TTA^a^ and no emergency procedure)
Primarily admitted patients	250	46 (18 %)	85 % (39/46)	15 % (7/46)	83 % (188/227)
Transferred patients	74	46 (62 %)	83 % (38/46)	17 % (8/46)	38 % (28/74)

Emergency procedures include damage control thoracotomy, damage control laparotomy, packing of the pelvis, revascularization of a limb, intervention radiology, craniotomy, insertion of intracranial pressure monitor, thoracostomy, external fixation of fractures for hemostasis, endotracheal intubation and other surgical procedures that aimed at stabilizing airways, respiration and circulation

TTA^a^: trauma team activation

A total of 92 patients received an emergency procedure, and the most common procedure was endotracheal intubation, followed by pleural drainage and neurosurgical interventions (Table 3). Hemostatic emergency surgery was required in <5 % of patients.

### Undertriaged patients

The 37 patients who were undertriaged according to ISS had a median ISS of 19 (IQR 17, 25), and 8 died within 30 days (22 %).

Neurosurgical injuries (head and/or cervical spine injury) were present in 32 of the 37 patients, and 84 % of these injuries were severe or critical injuries (AIS 4 or 5). This was significantly different from patients admitted with TTA (*p* < 0.001). Among the 32 undertriaged patients with neurosurgical injuries, 14 (44 %) patients were operated with a craniotomy or insertion of an intracranial pressure bolt (Table 3).

Two patients had severe chest injuries (AIS 4), there were no other injuries classified as AIS 4 or 5 below the neck.

Undertriaged patients were more often transferred from another hospital rather than admitted directly from the scene of accident (14 out of 37), compared to other included patients (*p* < 0.05).

### Triage criteria

The performance of the individual criteria is shown in Table 1. Several criteria are used in less than 5 % of the admissions, but with a relatively high fraction of correct triage (criteria number 1, 3, 4, 6–8, 11–17, 20).

## Discussion

Of the 131 seriously injured patients admitted at UNN and included in the present study, only 94 were admitted with TTA. This gives an undertriage of 28 and an overtriage of 74 % evaluated with extent of injury as the standard of reference (ISS). With occurrence of emergency procedures as reference, the undertriage was 15 and the overtriage 83 %. Based on the data from the 2011 study, we revised the TTA criteria and reinforced the transfer protocol. The present re-evaluation of our TTA protocol shows no significant change in over- or undertriage compared to the 2011 study.

Overuse of the TTA is a matter of resource utilization, and the only reason to accept overtriage is that some overtriage might be necessary to avoid undertriage. Our results are in line with several other studies that report an overtriage of ca 70 % [5–9]. Some authors report overtriage as high as 90 % [16], and in this context our results may still be acceptable. Indeed, some overtriage may help to increase clearance of patients from the emergency department, and unnecessary members of the trauma team may be dismissed early after the initial survey. In hospitals with a low TTA frequency overtriage also represents a potential for training the trauma team, which may improve team performance with seriously injured patients for which the TTA may make an important difference.

The low number of patients makes it difficult to draw conclusions about individual criteria for TTA, and even though some criteria were seldom used, the proportion of correctly triaged patients was high. However, based on the findings in the present study, we suggest that further changes in the present TTA criteria will not help to reduce overtriage. Another way to limit unnecessary use of limited resources may be to introduce a two-tiered TTA, with a low threshold for mobilizing a smaller team based on MOI information from the prehospital services, and the full team only when alarming vital signs or anatomical injuries are reported. Reports indicate a reduction of resource use and undertriage with a two tiered TTA [17, 18]. However, this has not been addressed by the present study.

A low undertriage is important for a favorable patient outcome, and there are reports indicating increased mortality in undertriaged patients [8]. According to the ACS-COT, an undertriage of up to 5 % is acceptable [3]. Our results are far above this figure, and we believe that two factors might explain some of the undertriage. First, at the UNN trauma registrars continuously screen all admissions to the surgical departments and assess for inclusion in the trauma registry. This might contribute to a better detection of undertriaged patients compared to other centers. Second, referring and receiving doctors may have exchanged critical patient information and agreed that a patient has been adequately stabilized before transfer and a new TTA at the trauma center is not needed. This is formally violating the existing transfer protocol in our center, but may represent sound clinical judgment and can in part explain some of the undertriaged transfers.

Neurosurgical patients dominate the group of undertriaged patients that have been directly admitted from the scene. These were scored to an ISS > 15, but the mechanism of injury and physiologic status, including level of consciousness, did not fulfill any TTA criteria. It seems possible to have a significant head injury, without being identified as seriously injured based on clinical findings. A recent study has shown that elderly patients with traumatic brain injury might have a higher Glasgow Coma Score (GCS) than younger patients, making our GCS-based triage criteria less reliable [19].

We have experienced an unexpected reduction in severe trauma admissions over the last ten years in our trauma center (unpublished data). In the present study, we report a decrease in TTA from 382 in 2005–2006 to 287 in 2013–2014, and likewise a reduction in number of seriously injured patients (ISS > 15) from 161 to 131 [12]. The results in this study cannot explain this general reduction, but we believe that it is not related to change in inclusions of patients. The recent introduction of a local trauma registry secures an even more complete inclusion of relevant trauma patients than before, and it is unlikely that the registrars now are missing more severe trauma patients than in the former study. Interestingly, the 30-day mortality was relatively unchanged with 6.6 in the 2011 study and 5.6 % in the present publication, supporting the notion that the inclusion process has remained unchanged.

Traditionally TTA has been evaluated against the ISS, with an ISS >15 indicating severe injury. AIS and ISS is an anatomical grading of injuries, with good correlation to mortality. However, the scoring of injuries is retrospectively derived, most often after discharge or death of the patients. AIS and ISS are therefore not available as an aid in decision-making in the prehospital setting or for the trauma team at admission. Furthermore, the most precise TTA-criteria for correct triage is the physiologic criteria, and there is a small paradox in assessing physiologic TTA criteria against anatomic injury grading. Anatomic and physiologic parameters often, but not always, correlate well with each other after injury. For this reason, we also evaluated TTA against immediate use of emergency procedures that aim to secure vital functions in physiologically instable patients. The main reason for a TTA is to identify such potentially life-threatening instability, and to restore airway control, and adequate ventilation and circulation. We believe, as other authors [20, 21], that the traditional ISS-based evaluation of TTA appropriateness should be complemented by the clinically recognized need for stabilizing emergency interventions. When we evaluated TTA against immediate emergency procedures, we found a slightly better undertriage, but still a high overtriage.

The number of severely injured patients is low, and this explains the low number of emergency procedures we have found. Hence, it has not been justified to have dedicated trauma surgeons on call at the UNN. Instead, the center relies on the general surgeons on call, who do both elective and acute care surgery on a daily basis. This indicates that specific trauma training of the surgeons is necessary, since real life experience with emergency procedures is limited. The recently suggested revised national trauma plan for Norway describes hemostatic emergency courses as mandatory for surgeons and their teams in all hospitals admitting trauma patients, in order to compensate for the limited experience in trauma surgery [22].

The weakness of our study is the low number of patients, which precludes an analysis of individual TTA criteria. The sample size was given by the time period we collected data, and a further extension of the time would both delay the evaluation, and it could also introduce other variables that would make it impossible to evaluate the effect of the criteria revision. Furthermore, we believe that not all criteria are assessed for every admission; if one criterion is fulfilled, it has no consequence to record more criteria, as the trauma team will be activated by the first criterion alone. Another potential limitation is the theoretical possibility that the results are influenced by an over-grading especially in head injuries, but all ISS scoring was done by trained and authorized registrars. We therefore believe that this possibility is unlikely. The major strengths of the study are the prospective design and the complete inclusion of undertriaged patients.

## Conclusion

Both overtriage and undertriage remains high despite changes in TTA criteria aimed to improve TTA protocol precision. Both indicators are still higher than desired. The study indicates a lack in the TTA criteria’s ability to identify patients with severe head injuries. The number of potential lifesaving surgical procedures is low. The revision of TTA criteria has not improved triage, and further studies are needed to find better ways to improve it.
